# Stereotactic Body Radiotherapy Boost with the CyberKnife for Locally Advanced Cervical Cancer: Dosimetric Analysis and Potential Clinical Benefits

**DOI:** 10.3390/cancers14205166

**Published:** 2022-10-21

**Authors:** Jiaxiang Gao, Benhua Xu, Yibin Lin, Zhenhang Xu, Miaoyun Huang, Xiaobo Li, Xiaodong Wu, Yuangui Chen

**Affiliations:** 1 Department of Radiation Oncology, Fujian Medical University Union Hospital, Fuzhou 350001, China; 2 The School of Medical Technology and Engineering, Fujian Medical University, Fuzhou 350004, China; 3 Fujian Key Laboratory of Intelligent Imaging and Precision Radiotherapy for Tumors (Fujian Medical University), Fuzhou 350001, China; 4 Clinical Research Center for Radiology and Radiotherapy of Fujian Province (Digestive, Hematological and Breast Malignancies), Fuzhou 350001, China; 5 Departments of Gynecology, Fujian Medical University Cancer Hospital, Fujian Cancer Hospital, Fuzhou 350014, China; 6 Executive Medical Physics Associates, Miami, FL 33179, USA

**Keywords:** locally advanced cervical cancer, stereotactic body radiotherapy, CyberKnife, high-dose-rate, interstitial brachytherapy, radiobiology, NTCP

## Abstract

**Simple Summary:**

The recommended treatment for locally advanced cervical cancer (LACC) consists of chemoradiotherapy (CRT) followed by brachytherapy (BT). Although BT is considered a minimally invasive procedure, patients still suffer severe discomfort from it and risk uterine perforation. Dosimetric uncertainties are often inevitable due to anatomical variations and inconsistencies in applicator loadings. These issues prompted us to explore the use of stereotactic body radiotherapy (SBRT) as a viable alternative. It has been well described that the CyberKnife (CK), a robotic image-guided SBRT delivery system, is capable of producing rapidly fall-off dose gradients with submillimeter accuracy. The aim of this study was to compare the dose distributions and radiobiological effects of a CK-based SBRT boost and a BT boost. We found a tumor volume threshold target, below which the CK-based SBRT plan could result in significantly better target coverage, OAR sparing and radiobiological effects compared to the BT plan. With improved precision of target localization, a reduced PTV margin might increase the eligibility of patients to receive a CK-based SBRT boost after CRT, rather than BT. CK-based SBRT could be an alternative option for patients who are not candidate for BT.

**Abstract:**

(1) Aim: To compare the treatment plans of stereotactic body radiotherapy (SBRT) with CyberKnife (CK) and high-dose-rate (HDR) intracavitary/interstitial brachytherapy (IC/ISBT) and examine the feasibility of CK-SBRT as a viable alternative to BT in patients with locally advanced cervical cancer (LACC). (2) Methods: A BT plan of 28 Gy in four fractions delivered previously to 20 patients with LACC was compared with a CK plan based on the same CT images with structures delineation for BT. The SBRT treatment plan was further divided according to two different approaches, with the high-risk planning target volume (HR-PTV) defined by the high-risk clinical target volume (HR-CTV) without and with a 5 mm margin, which were named CK-CTV plan and CK-PTV plan, respectively. The dose distributions and dosimetric parameters of the target volumes and organs at risk (OARs) were recorded and compared for the three boost plans. Radiobiological metrics were calculated based on the EUD for the hybrid plans. Additionally, the relationship between tumor volume and tolerance doses for the OARs in the BT plan and CK-PTV plan was investigated. (3) Results: Target coverage was better with the CK plan than with the BT plan, as the D95%, D98%, HI and CI of the CK-CTV plan and CK-PTV plan were higher than those of the BT plan; an exception was the D50%. Similarly, the TCP of the target was also significantly in favor of the CK hybrid plans (*p* < 0.01). For the OARs, the CK-CTV plan was superior to the BT plan as regards the rectum D2cc, bladder D2cc and bladder Dmax. The CK-PTV plan could achieve dosimetric parameters comparable to those of the BT plan for OARs concerning the small residual tumor volume. The NTCP of the rectum for the WPI+CK-CTV plans was significantly lower than that of the WPI+BT plans (*p* < 0.01). (4) Conclusions: CK-based SBRT can achieve better target coverage, dose sparing for the OARs and radiobiological effects compared with the BT plan for tumors that are not excessively large. CK-based SBRT could be an alternative option to administer a radiation boost for patients with LACC.

## 1. Introduction

Cervical cancer is the fourth frequent malignancy in women globally and remains a leading cause of cancer death in developing countries. Approximately 604,000 new cases of cervical carcinoma were diagnosed worldwide and 342,000 people died of the disease in 2020 [1]. External beam radiotherapy (EBRT) with concomitant chemotherapy followed by brachytherapy (BT) is the recommended treatment of choice for locally advanced cervical cancer (LACC) [2]. With the demonstrated improvement in clinical outcomes, BT is a crucial component in the management of LACC [3]. However, the use of BT has progressively declined since the early 2000s due to the rapid development of high-precision EBRT [4,5,6]. In certain circumstances, the practicability of BT is compromised because of unfavorable anatomy, medical comorbidities and patient refusal to receive the procedure. Furthermore, BT is operator-dependent, and radiation centers with a low treatment volume tend to pursue alternative EBRT boost modalities [5,6]. Previously published studies concluded that the clinical outcomes were adequately admissible to consider an EBRT boost if patients could not undergo brachytherapy [7,8].

With the advent of radiation delivery and image-guiding techniques, stereotactic body radiotherapy (SBRT) can now deliver a substantially higher dose to defined target volumes and effectively protect organs at risk (OARs). In addition, compared with other EBRT boost techniques, a higher biologically equivalent dose can be achieved by SBRT [9]. Earlier studies indicated that SBRT could be similar to BT in clinical outcomes and minimal toxicities for patients with cervical cancer [10,11,12,13]. In a propensity-matched analysis based on the National Cancer Database, those who underwent the SBRT boost had equal overall survival rates when compared with patients who received the BT one [14], which indicates that SBRT is a promising alternative for patients who are not candidates for BT.

The CyberKnife (CK), a robotic-based SBRT delivery system, enables the target volumes to be irradiated preeminently and produces rapid fall-off dose gradients, with submillimeter accuracy. The prescribed doses for linac-based SBRT in LACC treatment mostly remained below the recommended biologically equivalent doses of 2 Gy per fraction (EQD2) of at least 85 Gy [7], whereas the CK-based therapy could closely mimic the dose distribution of BT [11,15]. Moreover, radiobiological metrics such as tumor control probability (TCP) and normal tissue complication probability (NTCP) can be assessed and provide a more robust comparison of the efficacy of different radiotherapy modalities. However, there is very little information in the current literature regarding plan quality and the radiobiological effects of a CK-based SBRT boost in LACC patients compared with BT.

Given the challenges of administering BT and the benefits of CK-based SBRT, this study aimed to further investigate the dosimetric and radiobiological feasibility of SBRT with CK as an alternative to BT for patients with LACC.

## 2. Materials and Methods

### 2.1. Patient Characteristics

Twenty patients with pathologically confirmed LACC and FIGO stage IIA to IVA diseases examined from May to September 2021 were retrospectively reviewed in this study. All protocols were reviewed and approved by the Research Ethics Committee of Fujian Medical University Union Hospital (No. 2022ky175). The median age of the patients was 54.5 years (range 39 to 72 years). The median values of high-risk clinical target volume (HR-CTV) was 78.8 cm^3^ (range 37.78–128.44) in the BT plans. The median values of high-risk planning target volume (HR-PTV) was 137.58 cm^3^ (range 80.05–206.54) in the CK plans. The patient characteristics are summarized in Table 1. The prescribed dose of whole pelvic irradiation (WPI) was 48.6 Gy in 27 fractions, using the volumetric-modulated arc therapy technique. A BT boost to the HR-CTV was given with the prescribed dose of 28 Gy in 4 fractions. All cases did not receive EBRT on the day of BT irradiation. The Nucletro microSelectron-HDR remote afterloading unit (Elekta Inc., Stockholm, Sweden) was used for BT delivery.

### 2.2. CT Simulation

The patients were asked to prepare for the simulation, which included having a full bladder and an empty rectum. All patients underwent local anesthesia before the implantation of the applicator. Nucletron standard tandem/ovoid (T/O) applicators and interstitial needles were used to deliver the IC/ISBT treatment. The bladder and rectum were kept away from the applicator by a radio-opaque gauze. After the insertion, patients with the tandem and ovoid applicators in place were immobilized in the supine position. Computed tomography (CT) images with a 2.5 mm slice thickness were acquired on a CT simulator (Brilliance Big Bore, Philips, The Netherlands). The scanning range was set from the third lumbar vertebra to 5 cm below the ischial tuberosity.

### 2.3. Brachytherapy Planning

The BT plans of 20 patients were confirmed and delivered. The simulation CT images were transferred to an Oncentra treatment planning system V4.3 (Elekta Inc., Stockholm, Sweden). The HR-CTV was contoured in accordance with the Gynaecological Groupe Europeen de Curitherapie and the European Society for Therapeutic Radiology and Oncology (GYN GEC-ESTRO) recommendations. The OARs, including rectum, bladder, sigmoid, were outlined according to the Radiation Therapy Oncology Group (RTOG) consensus atlas. Briefly, the bladder wall, sigmoid wall and rectal wall were delineated as the outer wall of the organs minus 3 mm for the inner wall, so that a shell structure was created. A total of 28 Gy in 7 Gy per fraction was prescribed to the HR-CTV. The dose volume constraints in this study followed the National Comprehensive Cancer Network clinical practice guidelines [16]. Although a new plan was generated for each individual fraction of BT for every patient, for comparison purpose, the total sum of the 4-fraction BT was created from each patient’s first plan.

### 2.4. CyberKnife Planning

For the 20 patients, CT images with structures delineation for BT were transferred to the CK MultiPlan planning system V4.6.1. (Accuray Inc., Sunnyvale, CA, USA). The prescribed dose of target volume was 28 Gy in 4 fractions. Two different SBRT treatment plans were generated by MultiPlan and were named CK-CTV plan and CK-PTV plan, respectively. In the CK-CTV plan, the HR-PTV was equal to the HR-CTV of the BT plan. Considering the setup and systematic error in EBRT and tumor motion, previous studies indicated that the margin of planning target volume (PTV) from the HR-CTV was 5 mm in LACC [9,13,17]. Hence, the HR-PTV was built from the HR-CTV of the BT plan with a margin of 5 mm in the CK-PTV plan. The DVH data from the BT plan were used as input parameters in the CK plan’s optimization process for the corresponding patient. Dose volume constraints for the different OARs were according to the report of the American Association of Physicists in Medicine (AAPM) Task Group 101 during CK plan optimization [18]. The maximum monitor unit restrictions per beam and per node were set to 800 and 1200, respectively. The dose was calculated by the Monte Carlo algorithm (high resolution: 512 × 512 × number of slices, with 1% uncertainty). The grid of calculation was 0.2 cm. The Iris variable aperture collimator was used. Inverse planning was performed to acquire the optimal dose distribution. Overall, three plans were generated for each patient, i.e., a BT plan, a CK-CTV plan and a CK-PTV plan.

### 2.5. Evaluation of the Treatment Plans

The target volume coverage was assessed by calculating the D_50%_, D_95%_ and D_98%_ (dose delivered to 50%, 95% and 98% of the target volume). The dose homogeneity and conformity of the target volume were assessed by the homogeneity index (HI) and the conformity index (CI). The HI is an objective tool to analyze the uniformity of dose distribution in the target volume. The HI is defined as

(1) $$\mathrm{HI}=\frac{\left(D_{2\%}-{\;D}_{98\%}\right)}{D_{50\%}}$$

where D_2%_, D_98%_ and D_50%_ are the doses to the 2%, 98% and 50% of the target volume, respectively [19].

The CI helps to assess the degree of congruence between prescription isodose and planning target volume. CI is defined as

(2) $$\mathrm{CI}=\frac{V_{t,\mathrm{ref}}}{V_{t}}\times \frac{V_{t,\mathrm{ref}}}{V_{\mathrm{ref}}}$$

where V_t,ref_ is the volume of the target covered by the reference isodose, V_t_ is the target volume, and V_ref_ is the volume covered by the reference isodose [20]. Regarding the OARs, the following dosimetric parameters were recorded. D_2cc_ (dose to a volume of 2cc), D_max_ (maximum point dose to the organ), V_15Gy_ and V_24.5Gy_ (volume of rectum receiving 15 Gy and 24.5 Gy), V_17.55Gy_ (volume of bladder receiving 17.55 Gy).

### 2.6. Calculation of EUD, TCP and NTCP

The dose–volume histograms (DVHs) of the tumor CTV and OARs were derived from the WPI and three different boost plans. The equivalent uniform dose (EUD) is used as an evaluation tool under the assumption that two plans with the same value of EUD are equivalent in their therapeutic effectiveness. The dose was converted to EQD2 based on the LQ model to calculate the EUD [21,22]. Because 2 patients received WPI in other hospital, the DVHs for the WPI plans of only 18 patients were used. The EUD for three boost plans were separately summed with the EUD for the WPI plans to generate the total EUD for the hybrid plans (WPI+BT, WPI+CK-CTV and WPI+CK-PTV plans).

(3) $$EUD={\left(\underset{i}{\sum }\left(V_{i}D_{i}^{a}\right)\right)}^{\frac{1}{a}}$$

where $V_{i}$ is the fraction of the target volume irradiated by a dose $D_{i}$, and a is a unitless model parameter that is specific to the normal structure or the tumor of interest.

The tumor control probability (*TCP*) and normal tissue complication probability (*NTCP*)were calculated based on the total *EUD* [22]:

(4) $$TCP=\frac{1}{1+{\left(\frac{TCD_{50}}{EUD}\right)}^{4\gamma 50}}$$

(5) $$NTCP=\frac{1}{1+{\left(\frac{TD_{50}}{EUD}\right)}^{4\gamma 50}}$$

where $TCD_{50}$ is the dose leading to a 50% chance of controlling the tumor, $TD_{50}$ is the tolerance dose for a 50% complication rate of the normal structure at a specific time interval, $\gamma_{50}$ is a specific parameter that describes the slope of the dose–response curve. The following parameter values were used. For the tumor: a=−13, $TCD_{50}=50\;\mathrm{Gy}$, $\gamma_{50}=2.5$. For the rectum: a=8.33, $TD_{50}=80\;\mathrm{Gy}$, $\gamma_{50}=4$. For the bladder: a=2, $TD_{50}=80\;\mathrm{Gy}$, $\gamma_{50}=4$.

### 2.7. Statistical Analysis

Statistical analyses were performed with the SPSS Statistics 25.0 (IBM Corp, Armonk, NY, USA). The dosimetric parameters of the three plans (BT, CK-CTV and CT-PTV) were compared in a two-way analysis of variance (ANOVA), followed by Bonferroni post hoc testing. The radiobiological model results were compared in a paired *t*-test. To investigate the relationship between CTV and tolerance doses of the OARs, a paired *t*-test was used to test for the differences between the BT plans and the CK-PTV plans. The graphs were implemented in Origin 2021b (Origin software Inc., Northampton, MA, USA). *p* < 0.05 was considered statistically significant.

## 3. Results

### 3.1. Target Volume Coverage

**Figure 1:**
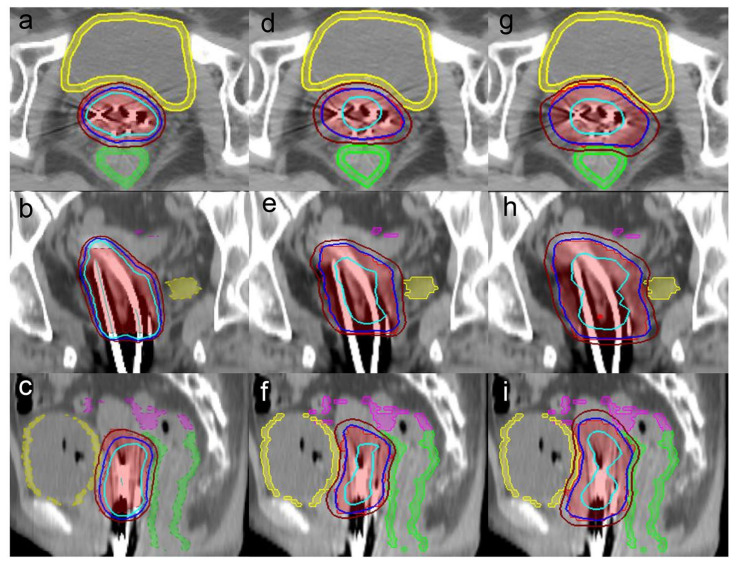
Axial, coronal and sagittal views of the dose distribution among the (**a**–**c**) BT plan, (**d**–**f**) CK-CTV plan and (**g**–**i**) CK-PTV plan for a representative patient. The 120% (33.6 Gy), 100% (28 Gy) and 80% (22.4 Gy) isodose lines are in light blue, dark blue, and brown, respectively. The images also illustrate the target volume in red, the rectum in green, the bladder in yellow and the sigmoid in pink.

**Figure 2:**
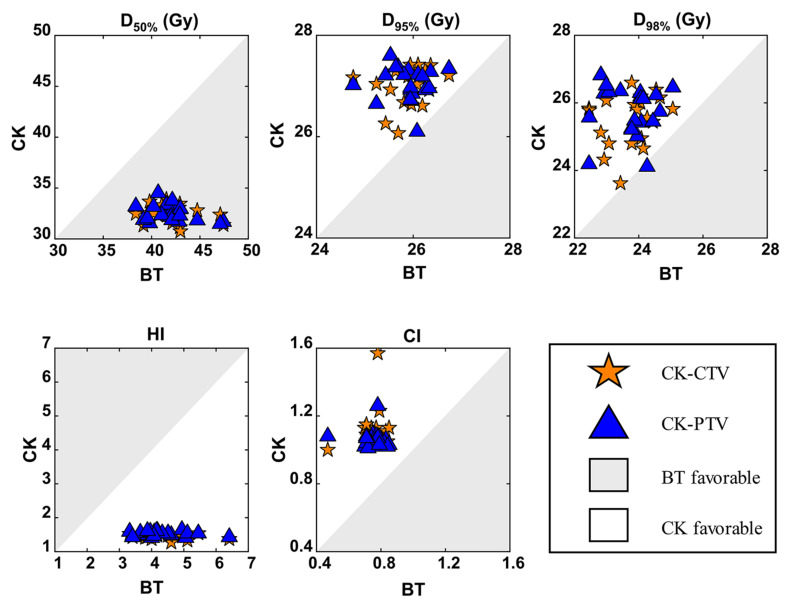
Comparisons of the BT, CK-CTV and CK-PTV plans by target volume dosimetric parameters. For every patient, each parameter is associated with three values (from the BT plan, CK-CTV plan and CK-PTV plan). In each graph, for example that of CI, each patient will contribute a pair of symbols, i.e., a star and a triangle; the star is plotted by the coordinates CI_BT_, CI_CK-CTV_, while the triangle is plotted by CI_BT_, CI_CK-PTV_. A symbol falling in the gray area implies the parameter is favorable in the BT plan. A symbol falling in the white area implies the parameter is favorable in the CK plan.

Target volume coverage in the CK-CTV plan and CK-PTV plan was superior to that in the BT plan. It was shown that the 120%, 100% and 80% isodose lines matched the tumor volume shape in the three plans (Figure 1). Except for D_50%_, the D_95%_ and D_98%_ of the CK-CTV plan and CK-PTV plan were higher than those of the BT plan (*p* < 0.001). Nevertheless, there were no statistically significant differences in all the dosimetric parameters between the CK-CTV plan and the CK-PTV plan (*p* > 0.05). The HI (*p* < 0.001) and CI (*p* < 0.001) were significantly better for the CK-CTV plan and the CK-PTV plan than for the BT plan, as the CK-CTV plan and CK-PTV plan showed significantly lower HI and CI than the BT plan, respectively. The results of the target volume parameters are presented in Table 2 and Figure 2.

### 3.2. Dose to Organs at Risk

**Figure 3:**
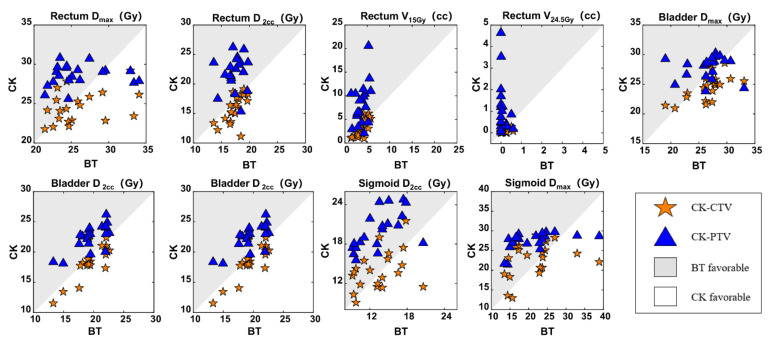
Comparisons of the BT, CK-CTV, and CK-PTV plans by the dosimetric parameters in relation to the OARs. For every patient, each parameter is associated with three values (from the BT plan, CK-CTV plan and CK-PTV plan). In each graph, for the parameter X, each patient will contribute a pair of symbols, i.e., a star and a triangle; the star is plotted by the coordinates X_BT_, X_CK-CTV_, while the triangle is plotted by X_BT_, X_CK-PTV_. A symbol falling in the gray area implies the parameter is favorable in the BT plan. A symbol falling in the white area implies the parameter is favorable in the CK plan.

A comparison of the dose parameters in relation to the OARs in the BT, CK-CTV and CK-PTV plans is presented in Table 3 and Figure 3.

#### 3.2.1. Rectum

The D_2cc_ was better with the CK-CTV plan (*p* = 0.019), whereas the D_max,_ V_15Gy_ and V_24.5Gy_ showed no statistically significant differences between the CK-CTV plan and the BT plan (*p* > 0.05). All rectum dosimetric parameters were inferior in the CK-PTV plan compared with the CK-CTV plan and BT plan (*p* < 0.05).

#### 3.2.2. Bladder

For the bladder, the D_2cc_ (*p* = 0.007) and D_max_ (*p* = 0.003) were significantly lower in the CK-CTV plan than in the BT plan; an exception was V_17.55Gy_. In addition, there were also statistically significant differences between the CK-CTV plan and the CK-PTV plan (*p* < 0.001), as the CK-CTV plan was superior to the CK-PTV plan in all dosimetric parameters. Except for D_max_ (*p* = 0.159), the D_2cc_ and V_17.55Gy_ were significantly lower in the BT plan than in the CK-PTV plan (*p* < 0.001).

#### 3.2.3. Sigmoid

The D_2cc_ and D_max_ showed no statistically significant differences between the CK-CTV plan and the BT plan (*p* > 0.05). Compared to the CK-PTV plan, the D_2cc_ (*p* < 0.001) and D_max_ (*p* = 0.002) were statistically significantly in favor of the CK-CTV plan. On the other hand, the D_2cc_ and D_max_ were lower with the BT plan compared to the CK-PTV plan (*p* < 0.001).

### 3.3. TCP and NTCP Analysis

The EUD and TCP of the target volume for the WPI+CK-CTV and WPI+CK-PTV plans were higher than those of the WPI+ BT plans (*p* < 0.001). The WPI+CK-CTV plans exhibited lower EUD and NTCP for the rectum, compared with the WPI+ BT plans (*p* < 0.001). For the bladder, the EUD and NTCP were comparable between the WPI+CK-CTV and WPI+BT plans. However, the EUD and NTCP of the OARs for the WPI+CK-PTV plan were higher than for the WPI+BT plans (*p* < 0.05). Table 4 shows the results of the comparison of the radiobiological metrics.

### 3.4. Relationship between CTV and Tolerance Doses for the OARs

We investigated the correlation between the size of the CTV and the quality of the CK-PTV. A threshold value of CTV was determined by using the paired *t*-test to screen the volumes of CTV from the largest to the smallest until no statistically significant difference in the OAR dosimetric indicators was shown between the BT and CK-PTV plans. For a CTV smaller than this threshold value, one could expect the CK-PTV plan to be comparable or better than the BT plan.

#### 3.4.1. Rectum

When the CTV was less than 65.55 cm^3^, no significant differences between rectum D_2cc_ and rectum V_24.5Gy_ were found between the BT plan and the CK-PTV plan, (*p* = 0.068 and *p* = 0.099). The maximum dose to the rectum was similar in the two plans when the CTV was less than 93.99 cm^3^ (*p* = 0.062). In addition, the rectum V_15Gy_ was comparable between the BT plan and the CK-PTV plan when the CTV was less than 75.52 cm^3^ (*p* = 0.081).

#### 3.4.2. Bladder

When the CTV was less than 56.50 cm^3^, there was no significant difference in the bladder D_2cc_ between the BT plan and the CK-PTV plan (*p* = 0.107). Moreover, the bladder D_max_ was similar for the BT plan and the CK-PTV plan when the CTV was less than 128.44 cm^3^ (*p* = 0.149). The bladder V_17.55Gy_ was comparable between the BT plan and the CK-PTV plan when the CTV was less than 57.99 cm^3^ (*p* = 0.051).

#### 3.4.3. Sigmoid

The sigmoid D_2cc_ was comparable between the BT plan and the CK-PTV plan when the CTV was less than 56.50 cm^3^ (*p* = 0.088). When the CTV was less than 75.52 cm^3^, there was no difference in the sigmoid D_max_ between the BT plan and the CK-PTV plan (*p* = 0.083).

When the CTV was less than 56.50 cm^3^, all of the OAR dosimetric parameters showed no statistically significant differences between the BT plan and the CK-PTV plan (*p* = 0.531 for rectum D_2cc_, *p* = 0.708 for rectum D_max_, *p* = 0.631 for rectum V_15Gy_, *p* = 0.635 for rectum V_24.5Gy_, *p* = 0.107 for bladder D_2cc_, *p* = 0.540 for bladder D_max_, *p* = 0.143 for bladder V_17.55Gy_, *p* = 0.088 for sigmoid D_2cc_, and *p* = 0.468 for sigmoid D_max_).

In summary, for patients with a CTV less than 56.50 cm^3^, the toxicity for all OAR was comparable between the BT plan and the CK-PTV plan, indicating that the CK SBRT is a viable alternative. The results are detailed in Table 5.

## 4. Discussion

Cervical cancer is one of the most prevalent cancers among females worldwide [23]. A combination of chemoradiotherapy and a BT boost is the standard of care for patients with LACC [24]. Although BT is considered a minimally invasive procedure, patients may suffer from vaginal pain, uterine perforation and anesthesia-associated risks [25]. Conversely, if patients are treated with SBRT, these risks could in principle be eliminated, with the added benefits of comfort and a shorter treatment time. Meanwhile, the SBRT allows a higher degree of dose control and a precise dose delivery to the cervical tumor volume, sparing the normal organs, compared to conventional radiation delivery methods. Therefore, many studies attempted to use SBRT to achieve a boost dose distribution similar to that of BT plans. Previously published results showed that SBRT could provide a potential alternative to boost cervical carcinomas when BT is not performed [15,26,27]. Moreover, Guerrero et al. suggested that SIB-IMRT was radiobiologically feasible for LACC patients who cannot undergo BT [28]. Unlike their study, our study not only presents a dosimetric comparison but also shows the achievement of the expected clinical outcome based on TCP and NTCP.

Earlier research demonstrated that a poor target coverage during radiation therapy is closely related to an increased risk of local and probably distant recurrence [29,30]. Our study indicates a consistent superiority of CK-based SBRT as a boost compared with BT in regard to target volume coverage and OAR sparing, and these results are similar to those previously published [11,26,31]. D_90%_ is considered the essential parameter for HR-CTV [32], but D_98%_ seems to be a better predictor of local control rates [33]. In our study, the CK-CTV plan showed statistically significant differences in the D_98%_ and D_95%_ when compared to the BT plan. D_98%_ and D_95%_ in the CK-CTV plan were higher than in the BT plan, while D_50%_ was on average 22.8% less than in the BT plan. That is to say that the results regarding the higher isodose volume outperformed those for the lower isodose volume. These findings are in line with the results of Malhotra et al. [34]. A study by Morgenthaler et al. [15] reported that the D_90%_ and V_100_ in the target volume were almost optimal when the boost was delivered by robotic radiosurgery. We also found that the EUD and TCP of the target volume for the WPI+CK-CTV plans were higher than those of the WPI+BT plans (*p* < 0.001). Additionally, the target volume coverage in the CK-PTV plan was superior compares to that of the BT plan in our study. With regard to plan quality, CK improved the dose homogeneity in the target volume. Our study showed that the HI and CI were also better with the CK-CTV plan and CK-PTV plan compared to the BT plan. The reason might be the geometrical variation and uncertainty of implantation in BT and, especially, the nature of the radiation from the BT source, resulting in a hot spot, a heterogeneous distribution and a suboptimal conformation of the prescribed dose.

The position of the cervix changes appreciably during the course of radiotherapy for cervical cancer. Previous studies examined the mean maximal inter-fractional movement of the cervix in the superior–inferior, anterior–posterior and right–left lateral dimensions and reported values of 2.1, 1.6 and 0.82 cm, respectively [35]. Moreover, cervix displacements greater than 1.5 cm occurred in intra-fractional radiation treatments [36]. Given organ motion and the uncertainties of the setup, HR-PTV was obtained from HR-CTV by a 5 mm margin in the CK-PTV plan in our study. It is notable that the CK-CTV plan was superior to the BT plan as regards rectum D_2cc_ (*p* = 0.019), bladder D_2cc_ (*p* = 0.007) and bladder D_max_ (*p* = 0.003) in our study. In addition, the EUD and NTCP values for the rectum were significantly lower in the WPI+CK-CTV plans (*p* < 0.001). For the bladder, the EUD and NTCP were comparable between the WPI+CK-CTV and WPI+BT plans. Cengiz et al. [11] compared the dose distribution characteristics in SBRT plans generated by a CK and in HDR BT plans for 11 patients with cervical cancer. No margin was added around the CTV to construct the PTV in the SBRT plans. Their study revealed distinct advantages in terms of target coverage and dose distributions to the surrounding normal tissues in SBRT plans, except for the bone marrow. The results are similar to ours with the CK-CTV plans. When the range of tumor motion is large, the internal target volume may be large, and this could cause increased the treatment toxicity [37]. However, the CK can achieve a submillimeter dose delivery with high precision, as tumor motion is tracked in real time using image guidance with different tracking system [38,39]. Hadi et al. assessed the feasibility of an MR-guided SBRT boost modality in patients who were ineligible for BT. Online-adaptive treatment planning was conducted to adjust the tumor volumes derived from daily anatomy [17]. If cervix motion is monitored or modified, it means that the margin of HR-PTV can be reduced. Hence, in order to minimize the margin of HR-PTV, it is suggested that adaptive radiotherapy planning be generated for each SBRT fraction, and real-time tracking and correction of cervix and tumor displacement be implemented. Such approach may result in a decrease in the volume of normal tissues exposed to the low-dose region, thereby reducing the late toxicity. Accordingly, dose distributions to the adjacent organs in a CK plan may be better than in a BT plan.

As has been previously revealed, the cervical tumor size is a critical independent prognostic factor for local control and pelvic recurrences after irradiation [40,41]. Promising clinical outcomes were reported by Mantz et al. [42], using a stereotactic ablative radiotherapy (SABR) boost in gross tumor volume (GTV) in 55 patients with FIGO stage IB–IIB cervical cancer. No grade 3 or greater late toxicities were observed, but this GTV-only modality needs to be further investigated. Albuquerque et al. [43] carried out a phase II trial of SABR as a boost for LACC, but the trial was closed due to severe toxicity concerns. The high rate of toxicity may be associated with the fact that large tumors were treated in this study. What accounts for late high-grade toxicity is the increased radiation exposure to OARs in the presence of bulky tumors. If the OARs are close to the PTV, the doses to the OARs may increase with a rise in the PTV [44]. Gultekin et al. retrospectively evaluated the oncological outcomes of an SBRT boost in 21 patients with cervical cancer. They found that while the 2-year LC rate was 75% in patients with residual tumor size < 4 cm, it was 50% when there was ≥4 cm residual tumor after definitive chemoradiotherapy (*p* < 0.001) [45]. It is suggested that a certain threshold of tumor size in patients with cervical cancer should be considered for eligibility to receive an SBRT boost. According to the size of the patient’s tumor, we arranged all dosimetric parameters in regard to the OARs and investigated the relationship between the tumor volume and OARs tolerance in the BT and CK-PTV plans. For the cohort of our study, when the tumor volume was less than 56.50 cm^3^, there was no statistical difference between the BT and the CK-PTV plans in all dosimetric parameters in relation to the OARs. This indicated that for patients with a target volume less than 56.50 cm^3^, lower OARs toxicity might be achieved with a CK boost. Systemic clinical trials are necessary for to provide a rigorous proof.

The main limitation of this study is that CT images with applicators were used to generate the CK plans, which is not in line with the actual treatment situation by CK. The applicators are not expected to be used in clinical treatments using the CK. The shape and location of the tumor will be affected during the implantation of the applicators. In a proposed future prospective study, a CT simulation without applicators will be performed after patients have received the last EBRT fraction. However, by the principle of physics, it is anticipated that the results of this study should hold true when no implanted applicators are in use.

## 5. Conclusions

Consistent with previous studies, CK-CTV plans can produce significantly better target coverage, OAR sparing, and radiobiological effects compared to BT plans. When the target volume is less than 56.50 cm^3^, CK-PTV plans with a 5 mm PTV margin can achieve a dose distribution comparable to that of BT plans. CK-based SBRT could be an effective alternative to BT for patients with LACC. With improved precision of target localization, a reduced PTV margin might increase the eligibility of patients with large tumors. Further clinical investigation to provide a higher level of evidence of the efficacy of a CK-based SBRT boost is needed.

## Figures and Tables

**Table 1:** Patients characteristics.

Variables	Value
Age (years) Median (range)	54.5 (39–72)
Histology (n)	
Squamous cell carcinoma	18
Adenocarcinoma	2
FIGO stage (n)	
IIA2	1
IIB	1
IIIA	1
IIIB	9
IIIC	6
IVA	2
Target Volume (cm^3^)	
HR-CTV (median, range)	8.8 (37.78–128.44)
HR-PTV (median, range)	137.58 (80.05–206.54)

FIGO, International Federation of Gynecology and Obstetrics; HR-CTV, high-risk clinical target volume; HR-PTV, high-risk planning target volume.

**Table 2:** Dose parameters comparison in relation to the target volume in the BT, CK-CTV and CK-PTV plans.

Index	BT	CK-CTV	CK-PTV	*p*-Value			
				ANOVA	a vs. b	a vs. c	b vs. c
D_50%_ (Gy)	42.08 ± 2.34	32.49 ± 0.87	32.53 ± 0.83	<0.001	<0.001	<0.001	1.000
D_95%_ (Gy)	25.89 ± 0.44	26.99 ± 0.39	27.05 ± 0.34	<0.001	<0.001	<0.001	1.000
D_98%_ (Gy)	23.74 ± 0.74	25.49 ± 0.77	25.75 ± 0.75	<0.001	<0.001	<0.001	0.849
HI	4.34 ± 0.75	1.47 ± 0.08	1.53 ± 0.07	<0.001	<0.001	<0.001	1.000
CI	0.75 ± 0.08	1.12 ± 0.12	1.06 ± 0.05	<0.001	<0.001	<0.001	0.147

**a**: BT; **b**: CK-CTV; **c**: CK-PTV. All values are shown as the mean ± standard deviation. D_n_%, dose delivered to n% of the target volume; HI, homogeneity index; CI, conformity index.

**Table 3:** Dose parameters comparison in relation to the OARs in the BT, CK-CTV and CK-PTV plans.

Index	BT	CK-CTV	CK-PTV	*p*-Value			
				ANOVA	a vs. b	a vs. c	b vs. c
Rectum D_2cc_ (Gy)	17.36 ± 1.59	15.84 ± 2.32	21.92 ± 2.78	<0.001	0.019	<0.001	<0.001
Rectum D_max_ (Gy)	26.02 ± 3.86	24.59 ± 2.06	28.57 ± 1.34	<0.001	0.166	0.004	<0.001
Rectum V_15Gy_ (cc)	3.71 ± 1.24	3.16 ± 1.80	8.30 ± 4.32	<0.001	1.000	<0.001	<0.001
Rectum V_24.5Gy_ (cc)	0.11 ± 0.18	0.03 ± 0.06	1.08 ± 1.17	<0.001	1.000	<0.001	<0.001
Bladder D_2cc_ (Gy)	19.64 ± 2.52	18.45 ± 2.79	22.48 ± 2.13	<0.001	0.007	<0.001	<0.001
Bladder D_max_ (Gy)	26.59 ± 3.25	24.40 ± 2.08	27.83 ± 1.90	<0.001	0.003	0.159	<0.001
Bladder V_17.55Gy_ (cc)	4.91 ± 2.89	5.09 ± 3.97	10.23 ± 4.03	<0.001	1.000	<0.001	<0.001
Sigmoid D_2cc_ (Gy)	13.44 ± 3.31	13.98 ± 3.04	20.00 ± 2.96	<0.001	1.000	<0.001	<0.001
Sigmoid D_max_ (cc)	21.14 ± 7.02	22.65 ± 4.39	27.44 ± 2.34	<0.001	0.760	<0.001	0.002

**a**: BT; **b**: CK-CTV; **c**: CK-PTV. OARs, organs at risk; D2cc, dose to a volume of 2cc; Dmax, the maximum point dose to the organ; Vn, volume receiving at least an n dose.

**Table 4:** Radiobiological comparison of CTV and OARs in the WPI+BT, WPI+CK-CTV and WPI+CK-PTV plans.

Index	WPI+BT	WPI+CK-CTV	WPI+CK-PTV	*p*-Value	
				a vs. b	a vs. c
CTV EUD (Gy)	84.50 ± 2.79	92.51 ± 3.18	93.64 ± 1.90	<0.001	<0.001
CTV TCP (%)	99.45 ± 0.19	99.77 ± 0.10	99.81 ± 0.04	<0.001	<0.001
Retum EUD (Gy)	74.70 ± 4.66	68.44 ± 3.86	77.65 ± 4.19	<0.001	0.021
Rectum NTCP (%)	28.08 ± 17.49	9.50 ± 6.17	39.38 ± 18.27	<0.001	0.035
Bladder EUD (Gy)	57.51 ± 3.22	57.14 ± 3.30	61.82 ± 3.74	0.234	<0.001
Bladder NTCP (%)	0.66 ± 0.44	0.59 ± 0.34	2.20 ± 1.84	0.303	<0.001

**a**: WPI+BT; **b**: WPI+CK-CTV; **c**: WPI+CK-PTV. EUD, equivalent uniform dose; TCP, tumor control probability; NTCP, normal tissue complication probability.

**Table 5:** Relationship between CTV and tolerance doses for OARs in the BT and CK-PTV plans.

Index	CTV (cc)	BT	CK-PTV	*p*-Value
Rectum D_2cc_ (Gy)	65.55	16.70 ± 1.97	19.87 ± 2.84	0.068
Rectum D_max_ (Gy)	93.99	26.34 ± 4.07	28.37 ± 1.37	0.062
Rectum V_15Gy_ (cc)	75.52	3.12 ± 1.21	5.44 ± 2.81	0.081
Rectum V_24.5Gy_ (cc)	65.55	0.18 ± 0.25	0.51 ± 0.39	0.099
Bladder D_2cc_ (Gy)	56.50	17.40 ± 3.42	19.84 ± 1.57	0.107
Bladder D_max_ (Gy)	128.44	26.59 ± 3.25	27.83 ± 1.90	0.149
BladderV_17.55Gy_ (cc)	57.99	2.66 ± 2.26	6.30 ± 3.93	0.051
Sigmoid D_2cc_ (Gy)	56.50	14.16 ± 3.94	17.79 ± 1.96	0.088
Sigmoid D_max_ (Gy)	75.52	23.56 ± 6.85	27.80 ± 1.46	0.083

OARs, organs at risk; D2cc, dose to a volume of 2cc; D_max_, maximum point dose to an organ; Vn, volume receiving at least an n dose.

## Data Availability

The data and clinical information will be shared upon reasonable request to the corresponding author.
